# Aptamer-Based Detection of Disease Biomarkers in Mouse Models for Chagas Drug Discovery

**DOI:** 10.1371/journal.pntd.0003451

**Published:** 2015-01-08

**Authors:** Fernanda Fortes de Araujo, Rana Nagarkatti, Charu Gupta, Ana Paula Marino, Alain Debrabant

**Affiliations:** 1 Laboratory of Emerging Pathogens, Division of Emerging and Transfusion Transmitted Diseases, Center for Biologics Evaluation and Research, United States Food and Drug Administration, Silver Spring, Maryland, United States of America; 2 Molecular Signaling Section, Laboratory of Molecular Immunology, National Institute of Allergy and Infectious Diseases, National Institutes of Health, Bethesda, Maryland, United States of America; Northeastern University, United States of America

## Abstract

Drug discovery initiatives, aimed at Chagas treatment, have been hampered by the lack of standardized drug screening protocols and the absence of simple pre-clinical assays to evaluate treatment efficacy in animal models. In this study, we used a simple Enzyme Linked Aptamer (ELA) assay to detect *T. cruzi* biomarker in blood and validate murine drug discovery models of Chagas disease. In two mice models, Apt-29 ELA assay demonstrated that biomarker levels were significantly higher in the infected group compared to the control group, and upon Benznidazole treatment, their levels reduced. However, biomarker levels in the infected treated group did not reduce to those seen in the non-infected treated group, with 100% of the mice above the assay cutoff, suggesting that parasitemia was reduced but cure was not achieved. The ELA assay was capable of detecting circulating biomarkers in mice infected with various strains of *T. cruzi* parasites. Our results showed that the ELA assay could detect residual parasitemia in treated mice by providing an overall picture of the infection in the host. They suggest that the ELA assay can be used in drug discovery applications to assess treatment efficacy *in-vivo*.

## Author Summary

A marked decrease in incidence of Chagas Disease has been observed in the last decade achieved by vector control strategies; however, there are still geographical areas where the disease reaches endemic proportions. Due to high morbidity and disease burden, other avenues of Chagas control, such as vaccines and therapeutic agents need to be employed for comprehensive disease control and mitigation. As there are no vaccines available currently, two drugs (Benznidazole and Nifurtimox) have been the mainstay of treatment. However, these drugs produce multiple side effects and frequently lead to early termination of the treatment. In this study, we have successfully developed a new method to evaluate the presence of Chagas biomarkers in the plasma of infected drug treated mice. Our study shows that high biomarker levels in *T. cruzi* infected mice, after drug treatment, can indicate treatment failure. Our assay provides a global picture of parasitemia in the host and a positive result would thus suggest that the treated animals continue to harbor *T. cruzi* parasites somewhere in the body. This study provides a new method to test for *T. cruzi* infection and for assessing the effectiveness of treatment.

## Introduction

Chagas disease (CD) is prevalent across various countries in Central and South America, with 28 million people at risk of getting infected [1], [2]. The etiological agent of CD, *Trypanosoma cruzi*, has two life cycle stages in the infected host, an extracellular trypomastigote form that circulates in blood of infected individuals, and an intracellular amastigote form that is present in the infected tissues and organs, such as the heart [3], [4]. Parasite infection and disease progression typically presents an acute phase characterized by blood parasitemia followed by a lifelong chronic phase, when circulating parasites can no longer be detected by direct blood examination [5].

Only two drugs, Benznidazole (Bz) and Nifurtimox have been the mainstay of treatment since the 1960–70s [6], [7]. However, these drugs produce multiple side effects leading frequently to early termination of treatment [8]. Additionally, the presence of intracellular amastigotes in the tissues and organs, where drug levels may not reach therapeutic concentrations, act as reservoirs of infection in the host [9], [10]. It has been estimated that for chronic individuals>80% of treated patients do not demonstrate parasitological cure and in the absence of drug therapy, or in immuno-compromised patients, recurrence of parasitemia is common [11], [12]. In light of these issues associated with CD therapy, new and better drugs need to be developed.

Chagas drug discovery initiatives have been hampered by the lack of standardized drug screening protocols, for example, pre-clinical assays that can be used to evaluate cure in animal models [13]. As trypomastigote levels can fluctuate in blood, detection of *T. cruzi* amastigote DNA by PCR in organs of infected drug-treated mice is a definitive end-point assay for determining cure [14], [15]. Immunosuppressing drug treated animals and allowing sub-patent infection to present itself as blood parasitemia, detectable using microscopy or PCR is another end-point assay indicating drug treatment failure [16]. Recently, several drugs, such as Posaconazole, VNI and Fexinidazole have been shown to cure mice using these methods [14], [15], [17]. However, for successful drug discovery, the ability to rapidly screen several drugs or their derivatives *in-vivo* for structure activity relationship studies is critical. Thus, due to the technical complexities associated with tissue PCR and immunosuppression protocols that require euthanization, these assays are far from ideal for pre-clinical drug discovery efforts [13]. These technical and other economic considerations have resulted in a significant lack of investment by the pharmaceutical industry in developing new drugs for neglected tropical disease such as Chagas disease [18].

We have recently reported an aptamer-based, non-serological, non-PCR assay, to detect *T. cruzi* biomarkers circulating in the blood of infected mice [19]. Aptamers are short nucleic acids, such as DNA or RNA, that are selected from a random library to bind specific targets, using a method called Systematic Evolution of Ligands by Exponential Enrichment (SELEx) [20]. We utilized this method to develop aptamers against a complex target: the *T. cruzi* excreted secreted antigens (TESA) [19]. In the current report, we performed SELEx to isolate additional aptamers that could detect *T. cruzi* biomarkers in murine drug discovery models of CD.

## Materials and Methods

### Ethics statement

Animal care protocol was approved by the Center for Biologics Evaluation and Research Animal Care and Use Committee (Protocol ASP #2010-03) and experiments performed following NIH Animal Care and Use Committee guidelines.

### Mice and parasites

Five to seven week old female, Swiss and C57BL/6, mice were infected with 5000 and 1000 blood stage *T. cruzi* trypomastigotes, respectively [13], [19], [21]. Swiss mice infected with the *T. cruzi* Colombiana strain were representative of the acute phase model of CD [13]. C57BL/6 mice infected with the *T. cruzi* Colombiana strain were representative of the chronic phase model of CD [21]. Parasite levels were determined using light microscopy obtained via tail vein bleed [19], [22]. The myotropic Colombiana strain and the Y strain of *T. cruzi* were maintained by serial passage every 21 and 10 days, in Swiss mice, respectively. The Y, Colombiana and 0704 strains of *T*. *cruzi* were cultured *in-vitro* using 3T3 mouse fibroblast cells [19], [23]. Infected 3T3 cell culture supernatant was collected and concentrated to obtain the TESA fraction [19]. Protein extracts from parasites unrelated to *T. cruzi*, such as *Plasmodium falciparum* infected RBCs, promastigotes of *Leishmania donovani*, *Leishmania major* and *Leishmania infantum* were obtained as described earlier [19].

### Benznidazole (Bz) treatment of mice

Both infected and non-infected mice were injected intraperitoneally (IP) with 200 µl Benznidazole (Bz, Sigma) (100 mg per Kg body weight) diluted in 50% DMSO in PBS, for 20 consecutive days [13]. After the 20 day treatment, mice were allowed to recover for 20 days and then bled and euthanized for collecting heart and skeletal muscle samples. Data presented are from one experiment, representative of three independent experiments, performed for each of the two drug treatment models.

### Aptamer selection against *T. cruzi* TESA

Aptamers were selected using an oligonucleotide pool Round 10 (R10) described previously [19]. Conditions used for each additional round of SELEx, from round 11 (R11) to round 21 (R21) have been described in S1 Table. Briefly, 100 µM aptamer pool was incubated at 65°C for 10 minutes and allowed to refold at room temperature for 1 hour. The refolded aptamer pool was then diluted to 1 ml in PBS, filtered through a 0.22 µm nitrocellulose (NC) membrane. The filtrate was then used for SELEx as described in S1 Table. Aptamer pool obtained at the last round of SELEx was cloned and biotinylated monoclonal aptamers and pools produced as described earlier [19], [23].

### Enzyme Linked Aptamer (ELA) assay

ELISA plates were coated with 50 µL of 2.5 µg protein/well of TESA, trypomastigote or epimastigote extracts or with mice plasma samples diluted 1∶200 in PBS [19]. Coated plates were blocked with 1% bovine serum albumin (BSA) in PBS. After discarding the blocking buffer, biotinylated RNA aptamers were added to each well. After 1 hour incubation the plate was washed thrice with PBS to remove unbound aptamers. Streptavidin-alkaline phosphatase was added to the wells and bound conjugate detected using 4-Methyllumbelliferyl Phosphate (4-MUP) (Liquid Substrate System, Sigma) [19]. Fluorescence was measured at an excitation wavelength of 360 nm and emission wavelength of 440 nm, with a cutoff filter of 435 nm, using a Spectra Max, M5, (Molecular Devices) [19].

### DNA extraction

The extraction of total genomic DNA from blood and tissue of animals infected with *T. cruzi* was performed using commercial kits, QIAamp DNA blood Mini kit, Qiagen and DNeasy Blood & Tissue Kit, respectively. Whole blood (50 µl) was first lysed with 1400 µl of 5% Saponin (Sigma) prepared in PBS. The lysate was centrifuged at 5000×g and the DNA isolated from the pellet containing the unlyzed cells, including parasites.

### Real time PCR assay

The PCR reactions were performed using 50 ng of DNA obtained from genomic tissue and 1 µl for DNA solution obtained from blood. The reaction mixture contained 0.4 µM of forward and reverse primers (F: 5′-AGTCGGCTGATCGTTTTCGA-3′; R: 5′-AATTCCTCCAAGCAGCGGATA-3′), 1× Premix Ex Taq (2× premix -Takara System) and 0.3 µl of SYBR Green (from a 100× stock, Invitrogen). For tissue PCR the genomic region encoding the IL12 p40 was utilized as the control to demonstrate equal amounts of purified DNA were used for all PCR amplifications. The genomic IL-12 p40-specific primers utilized were 5′-GTAGAGGTGGACTGGACTCC-3′ and 5′-CAGATGTGAGTGGCTCAGAG-3′ [21]. PCR was performed using the Bio-Rad CFX real time PCR machine with cycling conditions as follows: 95°C for 30 seconds; 95°C for 5 seconds and 64.2°C for 30 seconds, for 45 cycles. Amplification was immediately followed by a melt analysis to confirm specific amplification of parasite DNA [19], [23], [24].

### Statistical analyses

Data was plotted and statistical analysis carried out using GraphPad PRISM. Unpaired t-test with Welch correction was performed with a 95% confidence interval and p-values calculated. The ELA assay cutoff was calculated from the formula: mean RFU of control group (i.e., the non-infected drug treated group) + (2× standard deviation).

## Results

### Selection of RNA aptamers against *T. cruzi* excreted secreted antigens

We have recently reported the development of an Enzyme Linked Aptamer (ELA) assay to detect circulating *T. cruzi* Excreted Secreted Antigens (TESA) in the blood of the infected mice [19]. Eleven additional rounds of SELEx were performed under more stringent conditions with the starting library of round 10 (R10), obtained from our previous report, to increase the signal to noise ratio of the ELA assay (S1 Table) [19]. During each subsequent round of SELEx, the RNA aptamer pool was first depleted of non-specific aptamers, termed negative SELEx, by incubating the library with non-infected mouse plasma, and protein extracts from other parasites such as promastigotes of *L. donovani*, *L. major*, *L. infantum* and *P. falciparum* infected red blood cells (iRBC) (S1 Table).

**Figure 1:**
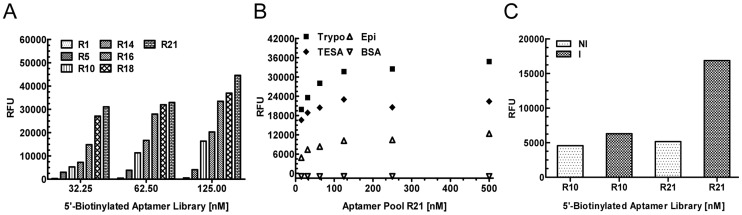
Binding analysis of aptamer pools at various rounds of TESA SELEx. **A**. Biotinylated aptamer pools obtained at various rounds of the TESA SELEx were used at 31.25, 62.50 and 125.00 nM concentrations in an ELA assay, plotted on the X-axis, to demonstrate binding to TESA. Relative fluorescence units (RFU) obtained are plotted on the Y-axis and each bar represents the mean of values from duplicate wells. Data shows a gradual enrichment of TESA binding aptamers from the starting SELEx library in round 1 (R1) to round 21 (R21). **B**. ELA assay was performed with serial dilutions of 5′-biotinylated R21 aptamer pool, plotted on the X-axis. The Y-axis represents aptamer binding to TESA in mean RFU for duplicate wells. The data shows a dose dependent saturable binding of aptamers to TESA, trypomastigote extract (Trypo), and epimastigote extract (Epi). There was no significant binding to Bovine serum albumin (BSA), used as a non-specific binding control. **C**. Biotinylated aptamer pools R10 and R21 at 100 nM, represented on the X-axis, were used to perform ELA assay with pooled plasma from non-infected mice (n = 4) and *T. cruzi* infected mice (n = 4). The RFU obtained were plotted on the Y-axis, with each bar representing the mean signal generated for duplicate wells. R21 aptamer pool showed approximately 2.5 fold higher signal in infected mouse plasma compared to R10 pool. Data shown is representative of at least three independent experiments.

ELA assay data with 5′-biotinylated aptamer pools from rounds 1, 5, 10, 14, 16, 18 and 21 indicated that, as the SELEx progressed, the proportion of TESA binding aptamers that constitute the pools from rounds R10 to R21 increased significantly (Fig. 1A). Compared to the R10 pool, R21 pool showed almost 6 fold increase in TESA binding signal (Fig. 1A). Aptamer pools R16, 18 and 21 showed binding at concentration as low as 32.25 nM, suggesting that they contained aptamers with higher binding affinities compared to aptamers obtained from R10. Dose response curve for the R21 pool showed saturable binding to TESA at concentrations ranging between 25 to 50 nM, thus indicating that the individual aptamers in this pool were high-affinity binders with minimal binding to the BSA control (Fig. 1B). The R21 aptamer pool also showed saturable binding to trypomastigote extract and at lower levels to *T. cruzi* epimastigote extracts (Fig. 1B).

Comparison of ELA binding data indicated that aptamers at R21 gave a significantly higher signal with the infected mice plasma compared to those at R10 (Fig. 1C). The signal to noise ratio (RFU Infected/RFU Non-Infected) improved from 1.3 for the R10 pool to 3.2 for the R21 pool. The higher stringency of SELEx conditions used from R10 to R21 improved the signal to noise ratio of the ELA assay, primarily due to enhanced binding characteristics of the R21 aptamer pool to plasma from *T. cruzi* infected mice (Fig. 1C).

### Monoclonal aptamers at Round 21 SELEx bind to *T. cruzi* TESA and trypomastigote lysates

**Figure 2:**
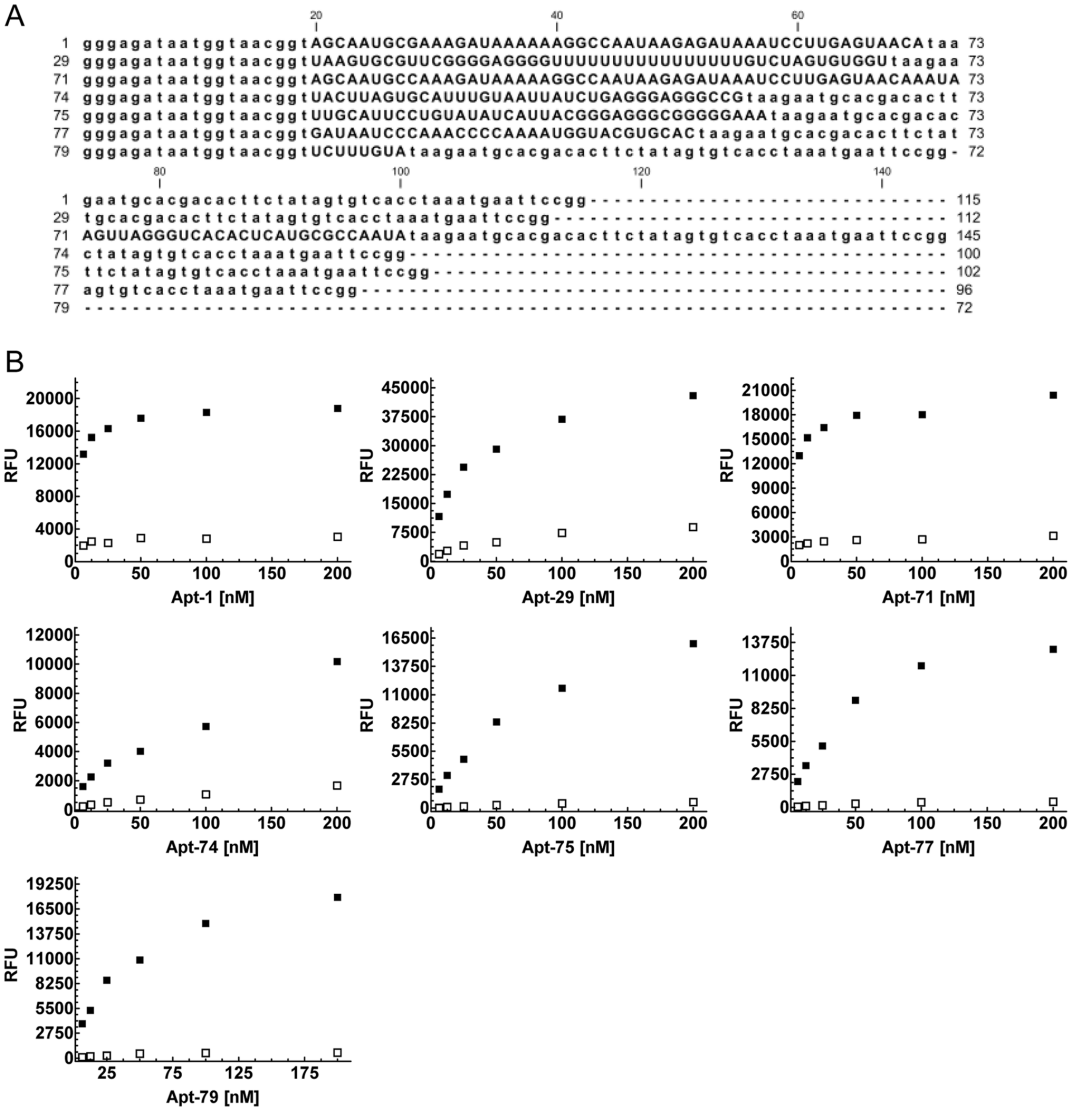
Sequence of monoclonal aptamers obtained at Round 21 TESA SELEx and binding to *T. cruzi* TESA preparations. **A**. RNA sequence, including the conserved T7 and SP6 primer binding sites (depicted in lower case) for the selected aptamers, Apt-1, 29, 71, 74, 75, 77 and 79 obtained from round 21 of TESA SELEx, are represented. The nucleotide length of each aptamer is indicated on the right. **B**. Serial dilutions from 200 nM to 6.25 nM of 5′-biotinylated monoclonal aptamers, Apt-1, 29, 71, 74, 75, 77 and 79, were used to perform ELA assays on polystyrene 96 well plates coated with 50 ng/µl/well, of TESA (filled squares) and BSA (open squares). Relative fluorescence units (RFU) obtained were plotted on the Y-axis and each point represents the mean of duplicate values. All the aptamers showed a dose dependent saturable binding to TESA but not to BSA. Data shown is representative of at least three independent experiments.

**Figure 3:**
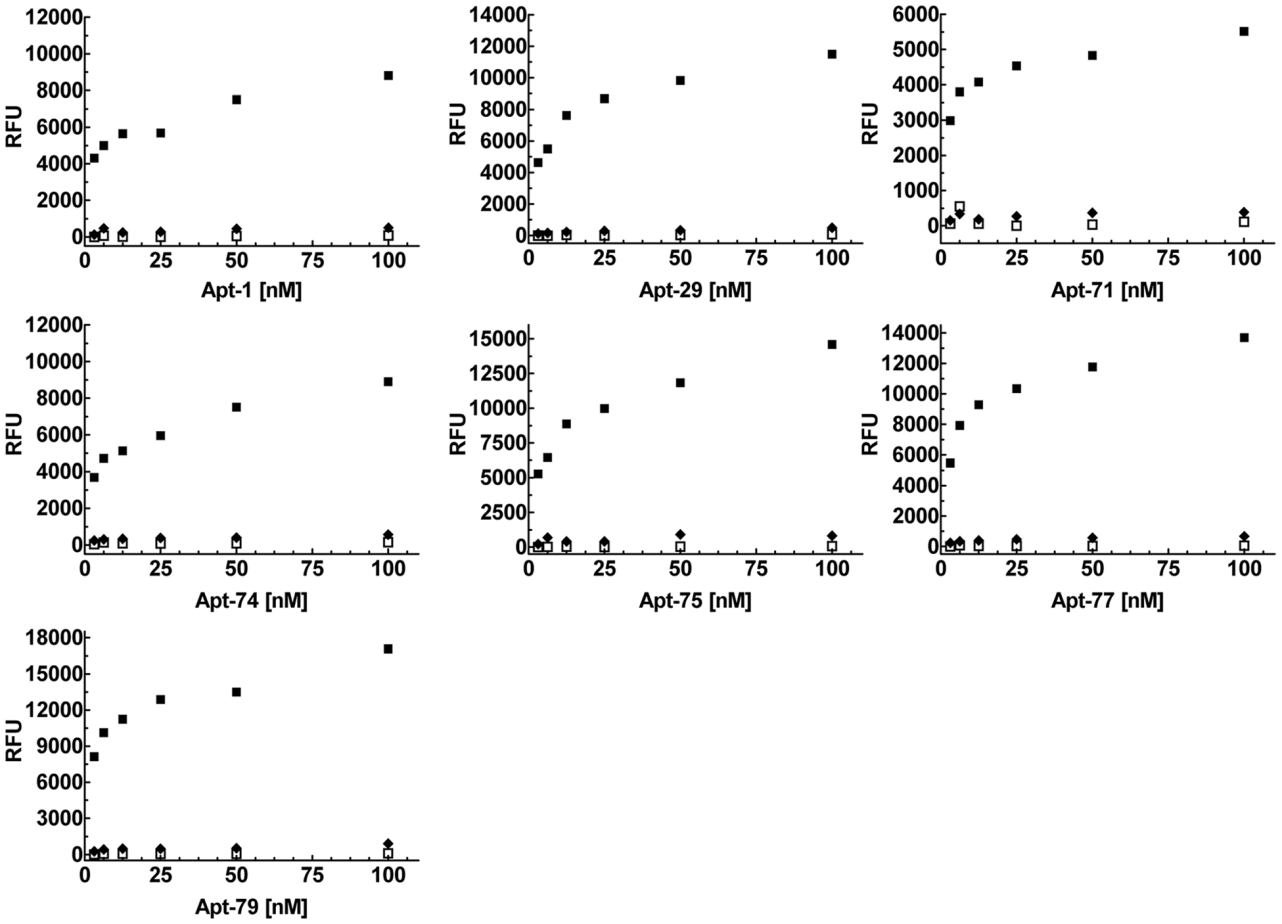
Selected monoclonal aptamers bind to *T. cruzi* trypomastigote protein extracts and not to epimastigote extracts or BSA. Serial dilutions from 100 nM to 3.125 nM of the seven 5′-biotinylated monoclonal aptamers, Apt-1, 29, 71, 74, 75, 77 and 79, were used to perform ELA assays on polystyrene 96 well plates coated with 50 ng/µl/well, of trypomastigote extract (filled squares), epimastigote extracts (filled diamond) and BSA (open squares). Relative fluorescence units (RFU's) obtained were plotted on the Y-axis and each point represents the mean of duplicate values. All the aptamers showed a dose dependent saturable binding to trypomastigote extracts and not to epimastigote extract or BSA. Data shown is representative of at least three independent experiments.

The R21 aptamer pool was cloned and sequenced to identify individual aptamer sequences. Phylogenetic analysis indicated that the sequences had converged to 7 major families, with a majority of the clones (>70%) belonging to Family 3 (S1 Fig.). The monoclonal aptamer sequences ranged from 72 to 145 base-pairs in length (Fig. 2A). A representative clone from each family was PCR amplified and used to generate 5′-biotinylated aptamers [19], [23]. All the selected biotinylated aptamers, Apt-1, 29, 71, 74, 75, 77 and 79, showed saturable binding to TESA but not to BSA which was used as a negative control (Fig. 2B). These aptamers also showed saturable binding to *T. cruzi* trypomastigote extracts (Fig. 3). Individual aptamers demonstrated higher signals with TESA than with equivalent amounts of trypomastigote protein extract (Fig. 3). This may be due to the higher relative abundance of their targets in the TESA fraction compared to the trypomastigote lysate. RNA Fold software, indicated that the 7 aptamers selected from R21 folded in highly stable three dimensional structures, formed by the various stem-loop regions of the sequences, with negative ΔG values ranging from −17.2 kcal/mol to −32.2 kcal/mol (S2 Fig.).

### Biomarker levels, detected by aptamers, predict drug treatment failure in an acute phase mouse model of CD

**Figure 4:**
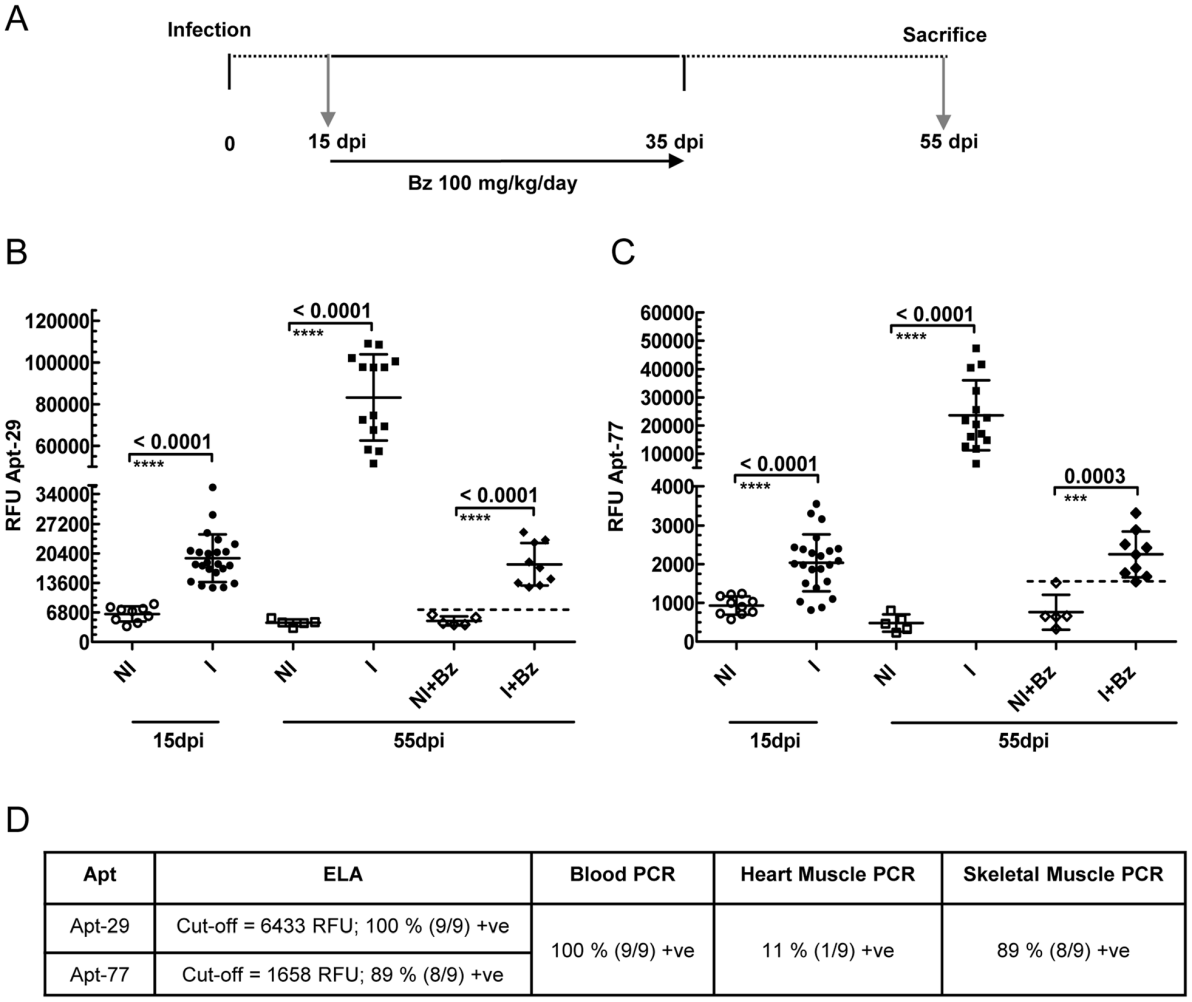
Mice treatment with Benznidazole (Bz) during Acute Phase. **A**. Schematic timeline of Bz treatment schedule of Swiss mice, infected with Colombiana strain of *T. cruzi*, during the acute phase of infection. Treatment was started at 15 days post infection (dpi) and continued till 35 dpi. The mice were bled and sacrificed at 55 dpi. The arrows represent the day blood samples were collected. Three independent drug treatment experiments were performed and representative data from a single experiment shown. **B**. Biomarker levels obtained by Apt-29 ELA assay in mice plasma before (15 dpi) and after (55 dpi) Bz treatment. **C**. Biomarker levels obtained by Apt-77 ELA assay in mice plasma before (15 dpi) and after (55 dpi) treatment. Results in **B** and **C** show the Relative Fluorescence Units (RFU), plotted on the Y-axis, for Apt-29 and Apt-77 biomarker detection by ELA assay, respectively, with lines depicting the mean values ±s.d. Differences in the mean values were considered significant for p<0.05. At 15 dpi the groups were labeled as non-infected (NI, n = 10) and infected (I, n = 23). At 55 dpi the groups are labeled as non-infected (NI, n = 5), infected (I, n = 14), non-infected treated (NI+Bz, n = 5) and infected treated (I+Bz, n = 9). **D**. Biomarker levels obtained by Apt-29 and 77 ELA assays in mice plasma in the acute phase (55 dpi) were used to calculate the number of mice still infected after Bz treatment, using the RFU cutoff values (mean of NI+Bz group +2×S.D.). *T. cruzi* DNA was detected in the blood, heart and skeletal muscles obtained from infected treated (I+Bz) mice at 55 dpi, by real time PCR. The non–infected control mice were all negative by real time PCR. The percentages of mice positive (+ve  =  positive) by ELA or PCR, were calculated by dividing the number of positive mice with the total number in the treated group and multiplying with 100.

A group of Swiss mice were infected with the Colombiana strain of the *T. cruzi* parasite and then treated with Benznidazole (Bz). Parasitemia was followed by microscopy and drug treatment was started at 15 days post infection (dpi) with 100 mg/kg/day Bz for a total of 20 days (Fig. 4A). Microscopy showed *T. cruzi* trypomastigotes in the blood of all the infected animals at 15 dpi. At 55 dpi the mice were sacrificed and blood, heart and skeletal tissues were collected for PCR and ELA assays to detect biomarkers. All seven aptamers, Apt-1, 29, 71, 74, 75, 77, and 79, were able to detect significantly higher levels of parasite biomarkers in plasma of infected mice compared to the age matched non-infected control mice at 55 dpi using the ELA assay (Fig. 4B–C and S3 Fig.). Further, all the aptamers, except Apt-1, showed significantly higher levels of biomarkers in the infected drug treated group compared to the non-infected drug treated controls (Fig. 4B–C and S3 Fig.). The non-infected drug treated group was utilized to detect any interference in the ELA assay by the drug alone and to establish the signal cutoff for the infected drug treated group (mean +2×s.d. of the non-infected treated group).

At 55 dpi, ELA assays with Apt-29 showed that the biomarker levels in 100% (9/9) of the infected drug treated mice were above the cutoff of the assay, suggesting that these animals could still be infected (Fig. 4D). Apt-29 ELA assay was as good as blood PCR (100% positive) at detecting mice that were still infected post drug treatment, but was significantly better than tissue PCR, as 11% and 89% of the infected treated group had detectable parasite DNA in the heart tissue and skeletal muscles, respectively (Fig. 4D). Similar results were observed with Apt-77 ELA, with 89% (8/9) of the mice showing biomarker levels greater than the cutoff (Figure 4C–D).

### Biomarker levels, detected by aptamers, predict drug treatment failure in a chronic phase mouse model of CD

**Figure 5:**
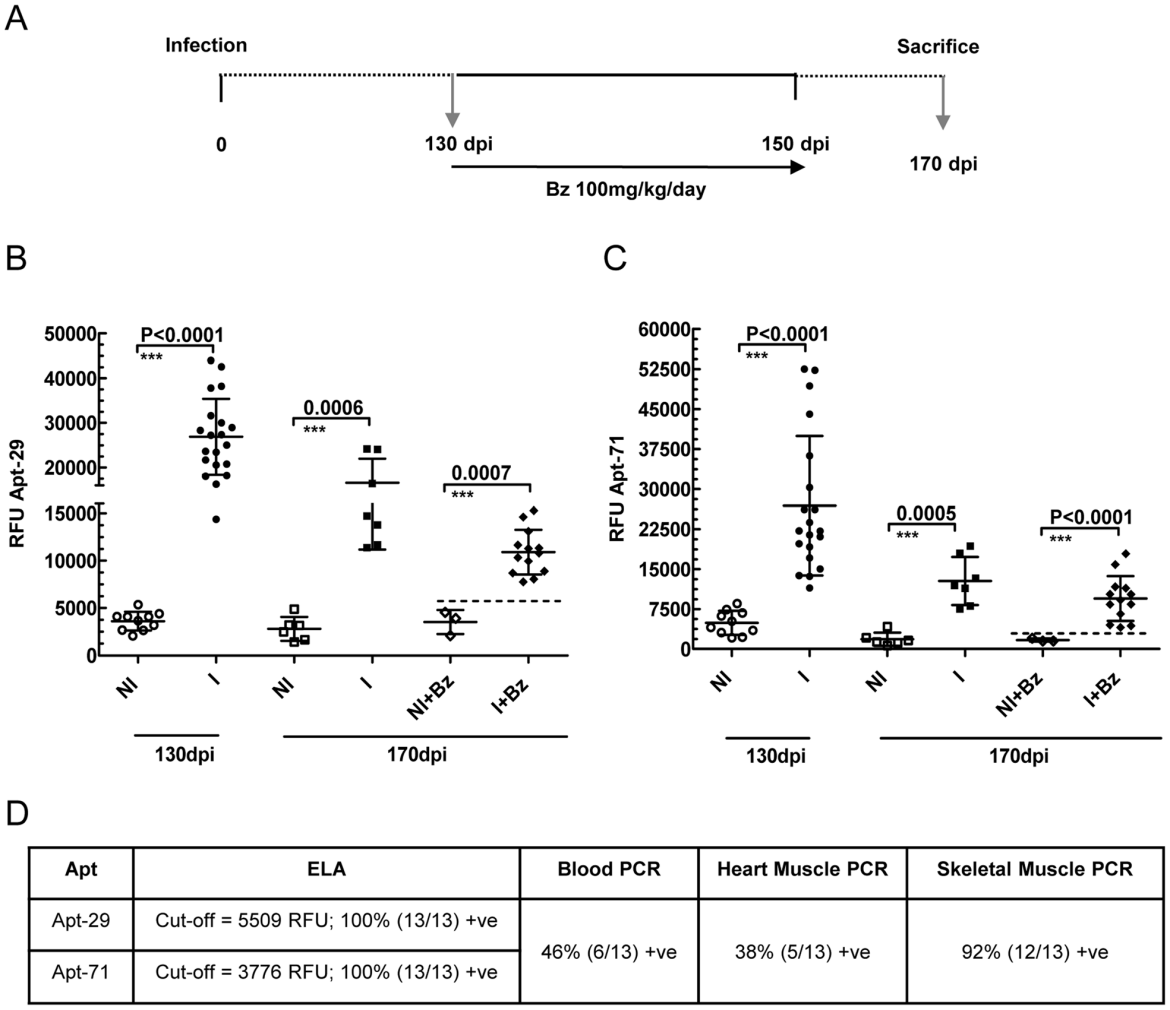
Mice treatment with Benznidazole (Bz) during Chronic Phase. **A**. Schematic timeline of Bz treatment schedule of C57BL/6 mice, infected with Colombiana strain of *T. cruzi*, during the chronic phase of infection. Treatment was started at 130 days post infection (dpi) and continued till 150 dpi. The mice were bled and sacrificed at 170 dpi. The arrows represent the day blood samples were collected. Three independent drug treatment experiments were performed and representative data from a single experiment shown. **B**. Biomarker levels obtained by Apt-29 ELA assay in mice plasma before (130 dpi) and after (170 dpi) treatment. **C**. Biomarker levels obtained by Apt-71 ELA assay in mice plasma before (130 dpi) and after (170 dpi) treatment. Results in **B** and **C** show the Relative Fluorescence Units (RFU), plotted on the Y-axis, for Apt-29 and Apt-77 biomarker detection by ELA assay, respectively, with lines depicting the mean values ±s.d. Differences in the mean values were considered significant for p<0.05. At 130 dpi the groups were labeled as non-infected (NI, n = 10) and infected (I, n = 20). At 170 dpi the groups are labeled as non-infected (NI, n = 6), infected (I, n = 7), non-infected treated (NI+Bz, n = 3), and infected treated (I+Bz, n = 13). **D**. Biomarker levels obtained by Apt-29 and 71 ELA assays in mice plasma in the chronic phase (170 dpi) were used to calculate the number of mice still infected after Bz treatment, using the RFU cutoff values (mean of NI+Bz group +2×S.D.). *T. cruzi* DNA was detected in the blood, heart and skeletal muscles obtained from infected treated (I+Bz) mice at 170 dpi, by real time PCR. The non–infected control mice were all negative by real time PCR. The percentages of mice positive (+ve =  positive) by ELA or PCR, were calculated by dividing the number of positive mice with the total number in the treated group and multiplying with 100.

To demonstrate that aptamers could also predict the outcome of drug treatment in a chronic phase model of CD, a group of C57BL/6 mice were infected with the Colombiana strain of *T. cruzi*. Drug treatment was started at 130 dpi with 100 mg/kg/day Bz for a total of 20 days until 150 dpi (Fig. 5A). At 170 dpi, all mice were sacrificed; blood, heart and skeletal tissues were recovered and analyzed by ELA assays and PCR. All seven aptamers were able to detect significantly higher levels of parasite biomarkers in plasma of infected mice compared to the age matched non-infected control mice at 170 dpi using the ELA assay (Figures 5B–C and S4 Fig.). Further, all the aptamers except, Apt-79 and 74, also showed significant difference between the level of biomarkers in infected drug treated group compared to the non-infected drug treated controls at 170 dpi (Figures 5B–C and S4 Fig.).

At 170 dpi, 100% (13/13) of the benznidazole treated mice were ELA positive using Apt-29 and 71 (Fig. 5D). In contrast, 46% (6/13) of the drug treated animals remained positive by blood PCR, 38% (5/13) by heart muscle PCR and 92% (12/13) by skeletal muscle PCR (Fig. 5D). Although one drug treated mouse was negative by blood and tissue PCRs, the internal control IL12 p40 was amplified from all these specimens and therefore the absence of *T. cruzi* DNA amplification was not due experimental errors. In this animal Apt-29 biomarker level was significantly higher (8047.61 RFU) compared to the assay cut-off (5509 RFU) and thus it was interpreted as infected (Fig. 5D). These results showed that Apt-29 and Apt-71 ELA assays were able to predict the failure of Benznidazole treatment accurately in 100% of the mice in this chronic mouse model.

### Biomarkers detection in mice infected with various strains of *T. cruzi* parasites

**Figure 6:**
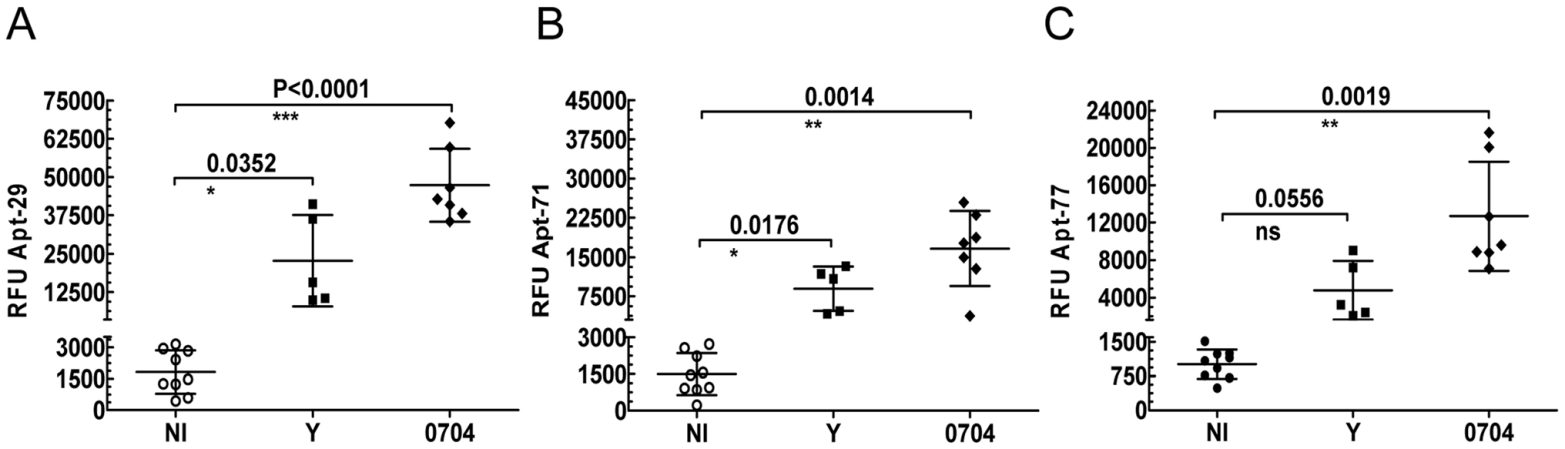
Aptamer binding to different strains of *T. cruzi*. Levels of parasite biomarkers detected during the chronic phase of infection, using Apt-29, 71 and 77 in ELA assays, are represented by the RFU's on the Y-axis with lines depicting the mean values ±s.d. Biomarker levels in plasma obtained from Swiss mice infected with the Y strain (n = 5) and 0704 strain (n = 7) of *T. cruzi* were compared with the non-infected group (n = 9) and differences in mean values considered significant for p<0.05.

To determine whether the aptamer-based biomarker detection assay could be used as a universal tool for anti-*T. cruzi* drug screening in murine models, we tested plasma obtained from mice in the chronic phase of infection with the Y and 0704 strains of the parasite. ELA assays performed with Apt-29, 71 and 77, showed that biomarker levels in these infected mice were significantly higher than the non-infected controls (Fig. 6). For Apt-77, although the biomarker levels in the Y strain infected mice were not statistically significant, a positive trend was observed (Fig. 6C). This data suggests that, the aptamers bind conserved targets across various strains of *T. cruzi*, the Tulahuen, used for SELEx, the Colombiana used for the acute and chronic phase models, and the Y and 0704 strains shown here, and that ELA assays could be used as a testing tool for anti-*T. cruzi* drug screening development using various murine models.

## Discussion

One of the primary reasons for failure to develop new drugs for CD has been the lack of standardized end-point assays in animal models [13]. The lack of universally accepted reliable tests to assess parasite burden and clearance and the hindrance this places on the development and evaluation of potential new drugs, both in experimental animal models and humans is well documented [25], [26]. In this study we describe a biomarker-based detection assay to assess treatment efficacy in murine drug discovery models for the screening of anti-*T. cruzi* drugs. The fact that aptamers bound to purified trypomastigote extract and that *T. cruzi* infected drug treated mice showed reduced levels of biomarkers, compared to the infected group alone, indicates that the targets of the aptamers were of parasite origin.

Current methods to assess cure in mouse models of CD are based on detecting parasite DNA in tissues samples or to immunosuppress drug treated animals and allow sub-patent infection to manifest as blood parasitemia that is detectable by microscopy or PCR [14], [15]. However, in the absence of immunosuppression, tissue PCR may give false negative results, for example, when sampling a part of infected tissue that may not have intracellular amastigotes. Additionally, various strains of parasites have varied tissue tropisms, particularly with different MHC restricted murine hosts [4], [27]. To overcome some of these issues, luciferase expressing parasites have been developed as tools for drug discovery [28], [29]. Recent data has demonstrated that *T. cruzi* parasites progressively migrate to, and infect different organs of the host, with the gastro-intestinal tract being the major reservoir of infection in the chronic phase [29]. Thus to demonstrate cure in these situations, all the tissues may have to be sampled by PCR, making such models laborious and impractical for drug screening purposes. Further, this limits drug screening to a few parasite strains that may be amenable to genetic manipulation. A biomarker-based assay, on the other hand, such as the one presented here, is based on the detection of parasite excreted secreted proteins in biological samples including serum or plasma. As parasite antigens secreted in different tissues, and transported via the interstitial fluids, will eventually be present in blood, the ELA assay provides a global picture of parasitemia in the host [30]–[33]. A positive ELA assay result would suggest that the treated animals continue to harbor *T. cruzi* parasites somewhere in the body thus indicating drug treatment failure.

Drug discovery models in the acute phase have been focused primarily on demonstrating the trypanocidal effect of the drug measured by the reduction or absence of parasites in blood, also termed parasitological cure, or massive reduction in parasite loads that could prevent further pathological damage [14]. However, most Chagasic patients are already in the chronic phase before their disease status is established and treatment can be initiated. In this regard, the BENEFIT trial is being conducted to determine the efficacy of treatment in the chronic phase [34]. The aptamers described in this report are able to detect parasite biomarkers in both the acute as well as the chronic phase of the disease.

As the animals do not need to be euthanized, a biomarker-based approach could yield continuous longitudinal data on the therapeutic effect of the drug. Moreover, ELA assays can be performed using standard ELISA equipment and do not require a highly controlled environment free of potential contaminating DNA, expensive reagents and complex instrumentation as PCR or flow cytometry. Another advantage of the aptamer-based assay is that these aptamers also detect targets in various strains of parasites, suggesting, that drug sensitive as well as drug resistant parasite strains, can be used in a biomarker-based drug screening protocol. It is worth noting here that the *T. cruzi* Colombiana strain is significantly more virulent and shows higher parasitemia in the mice strains used in this report compared to the Tulahuen or CL strain of the parasite, making it more difficult to cure [15], [35].

Another application where the ELA assay to detect biomarkers could be of use is the development of vaccines for CD [36]–[38]. Biomarker presence, detected by ELA assays, in parasite challenged vaccinated animals could indicate that the immune response was not sufficient to control the infection. The need for a reliable method to evaluate the cure of Chagas is a major concern, particularly when a clinical trial is designed. Furthermore, due to ethical reasons, commonly used protocols, such as tissue PCR and immunosuppression, cannot be used as endpoint assays for human clinical trials. The identification and validation of biomarkers for cure would be required before conducting human trials. Towards this eventual goal, further studies are being conducted to establish the utility of the reported aptamers for detecting biomarkers in samples from chagasic patients.

In conclusion, we have successfully developed aptamers that demonstrate high binding specificity and sensitivity to TESA. Moreover, we demonstrated the power of ELA assay to detect *T. cruzi* biomarkers in the plasma of infected mice and to predict drug treatment failure in both the acute and chronic phase murine models of Chagas disease. ELA assay can be used in drug discovery applications to assess drug efficacy *in-vivo*.

## Supporting Information

S1 Fig **Phylogenetic analysis of sequences obtained from Round 21 TESA SELEx.** Aptamer pool obtained at round 21 of the TESA SELEx was cloned into a TOPO cloning vector and 94 individual clones were isolated and sequenced. Aptamer sequences were analyzed using the Sequencher 2.4 software and aligned using the CLC Sequence Viewer 6.4 software. The Unweighted Pair Group Method using arithmetic averages (UPGMA) algorithm for distance data was employed to obtain converged families. Bootstrapping was performed with 1000 replicates and families obtained were labeled 1 through 7. A single clone from each family was selected for TESA binding studies.
(TIF)

S2 Fig **Predicted secondary structure of monoclonal aptamers selected from R21 TESA SELEx.** Predicted secondary structure obtained from Minimal Free Energy (MFE) calculations of the 7 aptamers are shown, with the calculated Gibbs free energy (ΔG, kcal/mol). The blue shaded sequence in the structures represent the conserved T7 and SP6 primer binding sites.
(TIF)

S3 Fig **Mice treatment with Benznidazole (Bz) during Acute Phase.** ELA assays were performed using Apt-1, 71, 74, 77, and 79, with mice plasma collected at 55 dpi. The groups were labeled as non-infected (NI, n = 5), infected (I, n = 14), non-infected treated (NI+Bz, n = 5) and infected treated (I+Bz, n = 9). Results show the relative fluorescence units (RFU), plotted on the Y-axis, for biomarker detection by ELA assay, with lines depicting the mean values ±s.d. Differences in the mean values were considered significant for p<0.05.
(TIF)

S4 Fig **Mice treatment with Benznidazole (Bz) during Chronic Phase.** ELA assays were performed using Apt-1, 74, 75, 77, and 79, with mice plasma collected at 170 dpi. The groups were labeled as non-infected (NI, n = 6), infected (I, n = 7), non-infected treated (NI+Bz, n = 3) and infected treated (I+Bz, n = 13). Results show the relative fluorescence units (RFU), plotted on the Y-axis, for biomarker detection by ELA assay, with lines depicting the mean values ±s.d. Differences in the mean values were considered significant for p<0.05.
(TIF)

S1 Table **Conditions utilized for performing SELEx with TESA.** Aptamers were selected from an oligo nucleotide pool R10 described previously [19]. For each iterative round of SELEx the amount of aptamer pool, TESA protein and conditions used have been described in S1 Table.
(DOCX)
