# Supplementary figures and images for: Aptamer-Based Detection of Disease Biomarkers in Mouse Models for Chagas Drug Discovery
Source: PLoS Negl Trop Dis. 2015 Jan 8;9(1):e3451. doi: 10.1371/journal.pntd.0003451 (PMC4287562; doi:10.1371/journal.pntd.0003451)

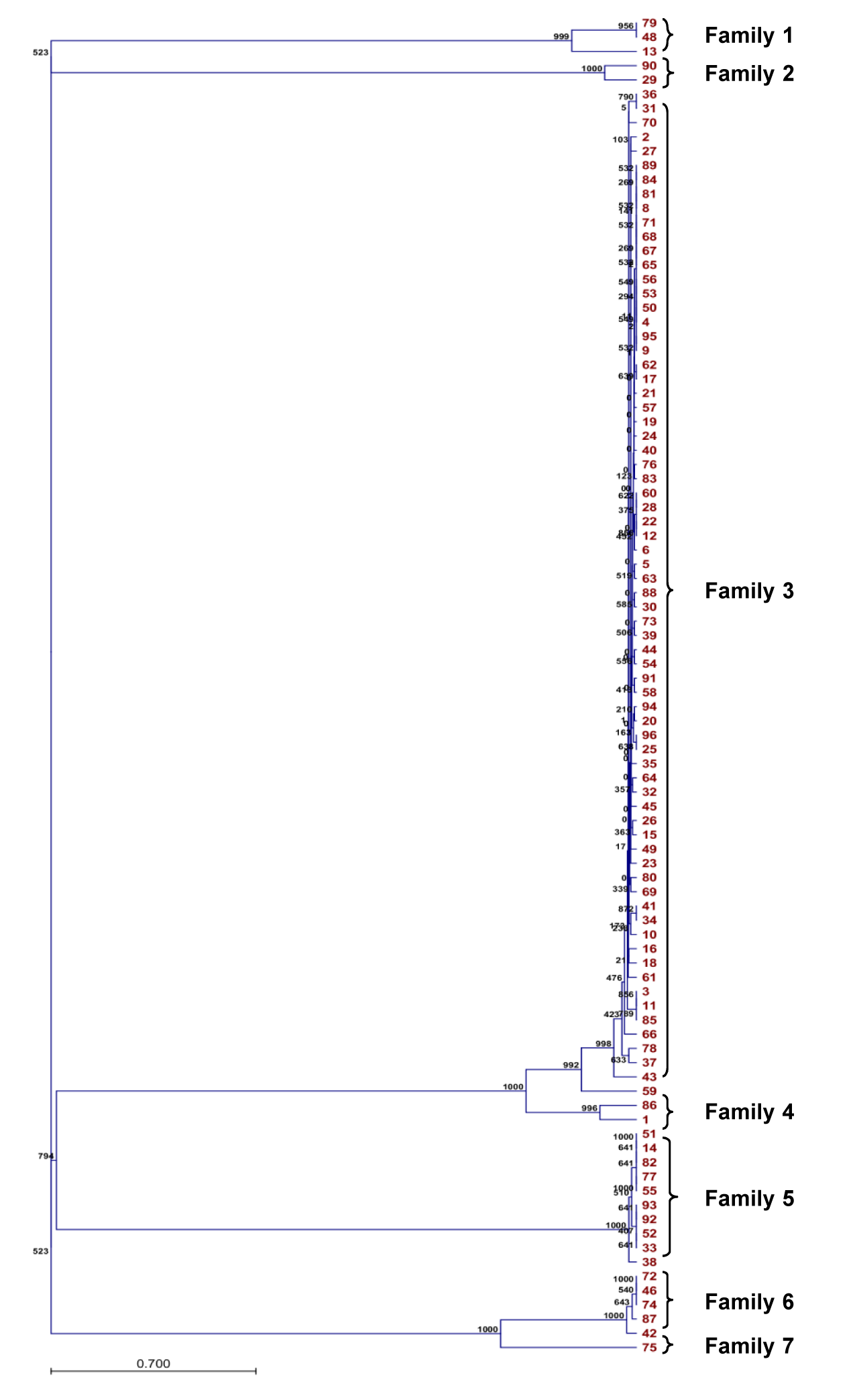

Supplement: S1 Fig — Phylogenetic analysis of sequences obtained from Round 21 TESA SELEx. Aptamer pool obtained at round 21 of the TESA SELEx was cloned into a TOPO cloning vector and 94 individual clones were isolated and sequenced. Aptamer sequences were analyzed using the Sequencher 2.4 software and aligned using the CLC Sequence Viewer 6.4 software. The Unweighted Pair Group Method using arithmetic averages (UPGMA) algorithm for distance data was employed to obtain converged families. Bootstrapping was performed with 1000 replicates and families obtained were labeled 1 through 7. A single clone from each family was selected for TESA binding studies. (TIF) [file pntd.0003451.s001.tif]

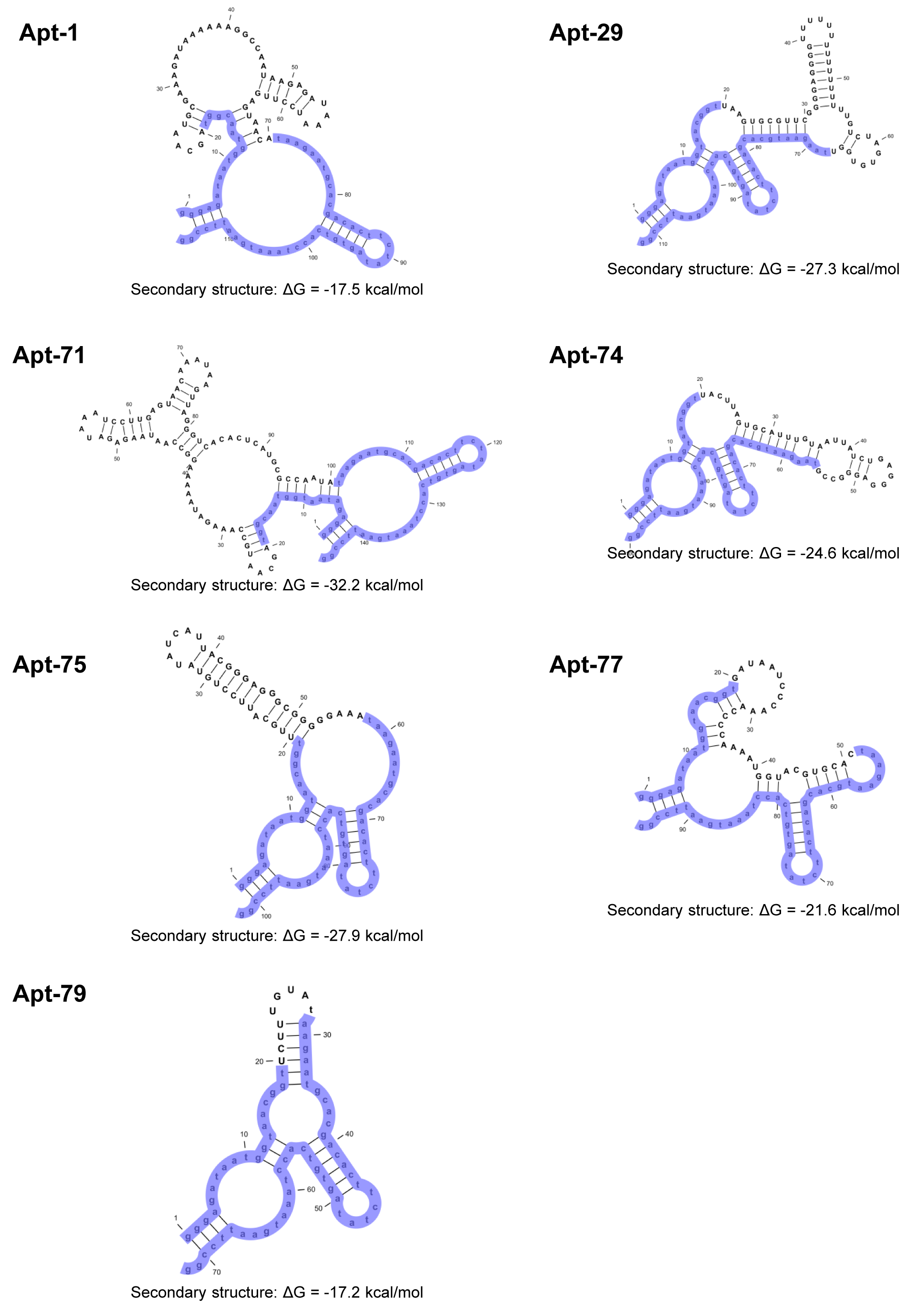

Supplement: S2 Fig — Predicted secondary structure of monoclonal aptamers selected from R21 TESA SELEx. Predicted secondary structure obtained from Minimal Free Energy (MFE) calculations of the 7 aptamers are shown, with the calculated Gibbs free energy (ΔG, kcal/mol). The blue shaded sequence in the structures represent the conserved T7 and SP6 primer binding sites. (TIF) [file pntd.0003451.s002.tif]

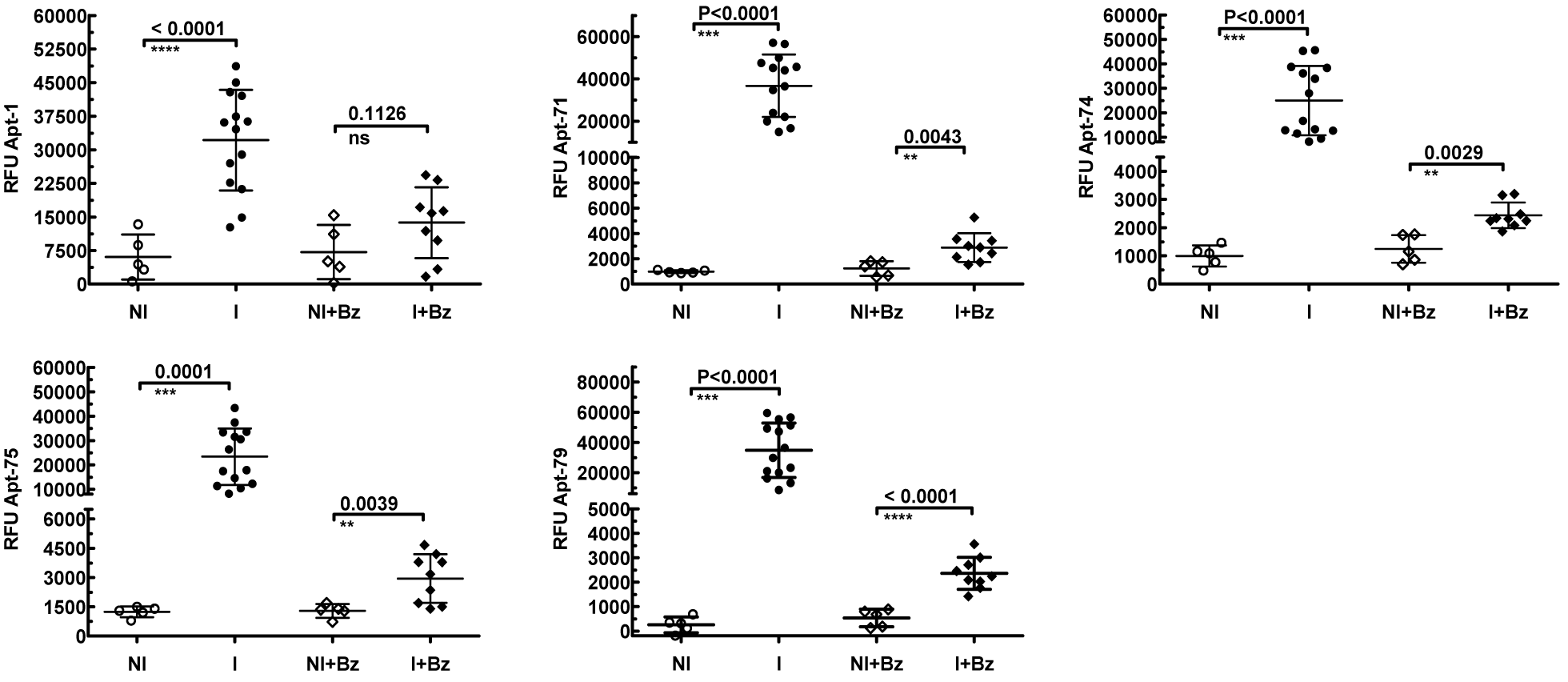

Supplement: S3 Fig — Mice treatment with Benznidazole (Bz) during Acute Phase. ELA assays were performed using Apt-1, 71, 74, 77, and 79, with mice plasma collected at 55 dpi. The groups were labeled as non-infected (NI, n = 5), infected (I, n = 14), non-infected treated (NI+Bz, n = 5) and infected treated (I+Bz, n = 9). Results show the relative fluorescence units (RFU), plotted on the Y-axis, for biomarker detection by ELA assay, with lines depicting the mean values ±s.d. Differences in the mean values were considered significant for p<0.05. (TIF) [file pntd.0003451.s003.tif]

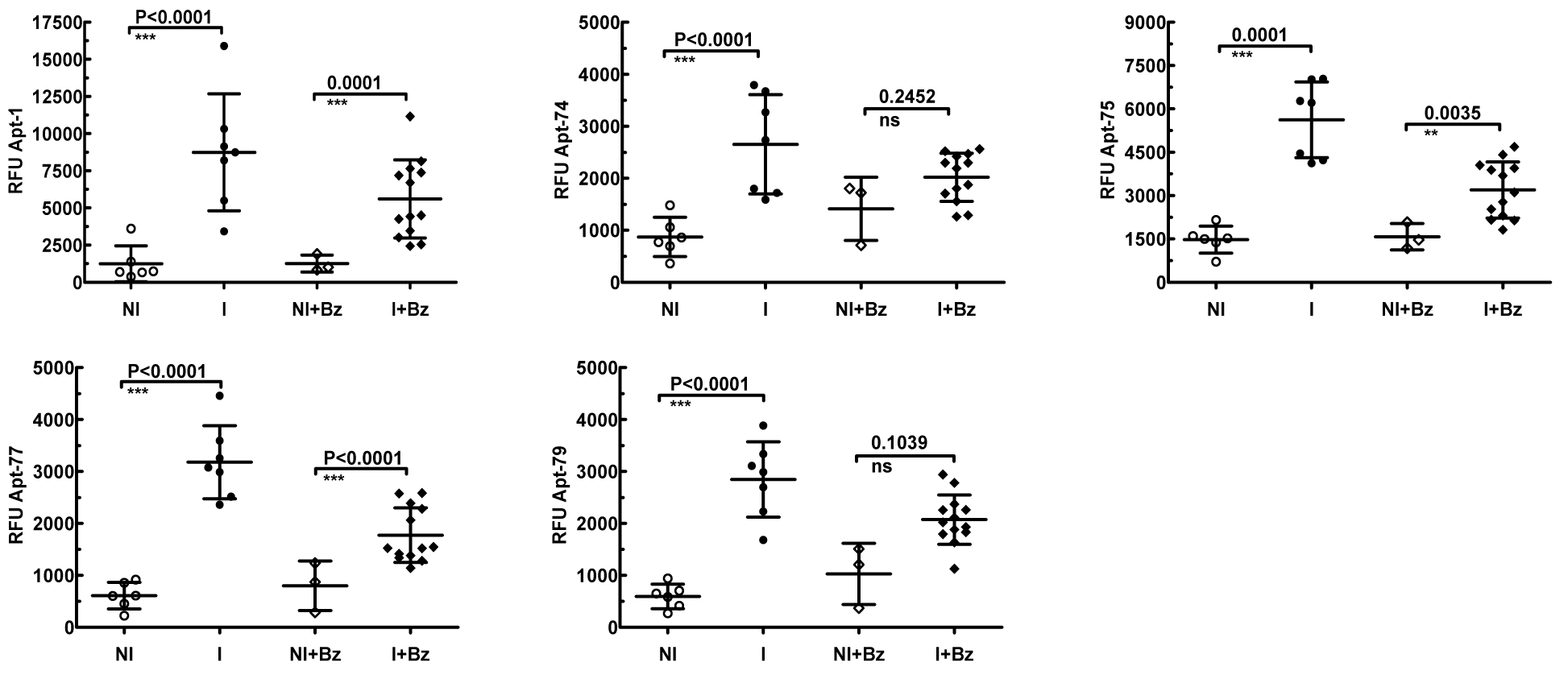

Supplement: S4 Fig — Mice treatment with Benznidazole (Bz) during Chronic Phase. ELA assays were performed using Apt-1, 74, 75, 77, and 79, with mice plasma collected at 170 dpi. The groups were labeled as non-infected (NI, n = 6), infected (I, n = 7), non-infected treated (NI+Bz, n = 3) and infected treated (I+Bz, n = 13). Results show the relative fluorescence units (RFU), plotted on the Y-axis, for biomarker detection by ELA assay, with lines depicting the mean values ±s.d. Differences in the mean values were considered significant for p<0.05. (TIF) [file pntd.0003451.s004.tif]
