# Supplementary material for: Aptamer-Based Detection of Disease Biomarkers in Mouse Models for Chagas Drug Discovery
Source: PLoS Negl Trop Dis. 2015 Jan 8;9(1):e3451. doi: 10.1371/journal.pntd.0003451 (PMC4287562; doi:10.1371/journal.pntd.0003451)
Supplement: S1 Table — Conditions utilized for performing SELEx with TESA. Aptamers were selected from an oligo nucleotide pool R10 described previously [19]. For each iterative round of SELEx the amount of aptamer pool, TESA protein and conditions used have been described in S1 Table. (DOCX) [file pntd.0003451.s005.docx]

| **SELEx Rounds** | ***SELEx Target*** | **RNA aptamer Pool** | **SELEx Conditions** |
| --- | --- | --- | --- |
| R11-13 | TESA 50 μg/ml | 100 pmol | Negative SELEX (NS): The aptamer pool was refolded at a concentration of 100 mM. The refolded aptamer was diluted to 1 ml in PBS and sequentially passed thru two reusable filter assembly with Nitrocellulose (NC) membrane filter (0.22 μm) to remove sequences that bind to NC. The volume was adjusted to 1 ml and non-infected plasma (at a final dilution of 1: 200 in PBS) and non*-T. cruzi* parasite protein lysate (at a final concentration of 100 μg/ml) was added to the aptamer pool. This solution was incubated for 30 minutes at room temperature and sequentially filtered thru 3 filter assemblies with NC membranes.  Positive SELEX (PS): Recovered filtrate from NS was adjusted to 1 ml with PBS and TESA added at a final concentration of 50 μg/ml. The solution was vortexed gently and incubated at room temperature for 30 minutes. The solution was filtered thru a NC membrane containing filter assembly. The membrane was washed by filtering 20 ml of PBS thru it. The NC membrane was recovered from the filter assembly and bound RNA aptamers were recovered using the Promega total RNA isolation kit. |
| R14-15 | TESA 50 μg/ml | 100 pmol | NS was performed as above for rounds 11-13, with a modification that the filtrate was sequentially passed thru 10 filter assemblies with NC membranes to capture any aptamer-protein complexes or aptamers that bind to NC.  PS was performed as for rounds 11-13, with the modification that after the aptamer pool was incubated with TESA, the solution was diluted to 20 ml, to increase selection pressure, before being passed thru the NC membrane filter. The NC membrane was washed by filtering 20 ml of PBS. Membrane bound RNA aptamers were recovered as above. |
| R16-17 | TESA 50 μg/ml | 100 pmol | NS was performed as above.  PS was performed as above with the modification that the 20 ml diluted aptamer-TESA solution was incubated at room temperature for 2 hours before filtration and RNA isolation. |
| R18-19 | TESA 50 μg/ml | 100 pmol | NS was performed as above.  PS was performed as above with the modification that the 1ml aptamer TESA solution was diluted to 50 ml PBS and incubated at room temperature for 2 hours before filtration and RNA isolation. |
| R20-21 | TESA 50 μg/ml | 100 pmol | NS was performed as above.  PS was performed as above with the modification that the 1ml aptamer TESA solution was diluted to 50 ml PBS and incubated at room temperature for 4 hours before filtration and RNA isolation. |
